# Dacarbazine nanoparticle topical delivery system for the treatment of melanoma

**DOI:** 10.1038/s41598-017-16878-1

**Published:** 2017-11-28

**Authors:** Abdul Hafeez, Imran Kazmi

**Affiliations:** Glocal School of Pharmacy, Glocal University, Mirzapur Pole, Saharanpur, Uttar Pradesh India

## Abstract

Dacarbazine (DZ) is poorly soluble in water with the short half-life in blood circulation, low rate of response with the toxic effect which ultimately limits its utilization of the treatment of skin cancer. In view of this background current study was designed for development of dacarbazine laden nanoparticle (DZNP) and dacarbazine laden nanocream (DZNC) topical delivery system for the treatment of melanoma. Firstly DZNP was prepared. By using DZNP its cream formulation prepared for topic drug delivery for melanoma. Dacarbazine nanoparticle and its cream were evaluated for morphology, drug load capacity, efficiency of nanoencapsulation and size of particle and zeta potential, Transmission Electron Microscopy (TEM), determination of pH, spreadability and viscosity, *in vitro* drug release capacity and its cytotoxic potential. The particle size of DZNP and DZNC was 16.3 ± 8.1 nm and 16.9 ± 7.8 nm respectively. pH value and spreadability of nanoparticle cream were found to be 6.7 ± 0.14 g cm/sec and 55.23 ± 3.13 g cm/sec respectively. Nanoencapsulation efficiency and Drug loading capacity were 67.4 ± 3.5% and 6.73 mg/10 mg respectively. IC50 of dacarbazine nanoparticle was 0.19 mg/ml while it was 0.63 mg/ml for nanoparticle cream. It can be concluded that DZNP and its cream can be effectively used as a topical formulation for the treatment of melanoma.

## Introduction

Skin cancer is the most common form of cancer and covers half of all new cancer cases in Western countries. The incidence of skin cancer is increasing considerably. In the United States, it is predicted that in future one out of five individual will suffer from cancer of skin in their lifespan^1^. Cancers of skin can be divided into two main classes, one is melanoma cancer and another one is nonmelanoma skin cancer.

Most severe and dangerous form of skin cancer is melanoma^2^. As per Estimates of American Cancer Society, at the end of 2017, it is estimated that 87110 new cases of melanoma will be diagnosed and out of which 9730 people will die from melanoma^3^. Detection of melanoma at an early stage would be helpful in treatment. Melanoma generates from the uncontrolled cell growth in the pigmented skin spots. It is the uncontrolled cell division of melanocytes which are found as a single layer of cells within the epidermal basal layer. It is severe form of cancer of skin which having the potential of metastasis and mainly responsible for the most of the deaths related to skin. Five year survival rate of metastatic melanoma is only around 10%^4^.

For the treatment of malignant form of melanoma, different methods have been utilized viz. Surgery, add-on therapy, radiation therapy and chemotherapy and immunotherapy^5–9^. For most of the melanomas surgery is the main option of treatment and give relief in early stage of melanomas^10^. When it is diagnosed by the biopsy of skin, extensive surgery required for complete removal of cancerous cells from skin^11^. In some cases, Mohs surgery required for the treatment. Mohs surgery is done by a special expert surgeon or dermatologist, in which thin layer of melanoma is removed along with the skin^12^. There is a very unlikely chance of cure from surgery, once melanoma has metastasized from the skin to other parts of the body like brain or lungs^13^. Melanomas with high-risk need add-on therapy along with main treatment. In adjunct therapy, interferon is used^14^. Most of the patients gain good health with the high dose of interferon treatment although it has severe adverse effects on the body. Chemotherapy agents like dacarbazine, temozolomide, and immunotherapy with interleukin-2 are used depending on severity of patients^15^.

Among the chemotherapeutic drug dacarbazine (DZ) is the single US-FDA approved anticancer drug, is now utilized as a drug of choice chemotherapy medicine against the melanoma cancer^16^. DZ is the one of the member of the class of anticancer drugs known as alkylating agents, which kills cancerous cells by the addition of an alkyl group to the DNA of cancer cell^17^. Moreover, usage of DC in the treatment of melanoma is restricted due to its various disadvantages. First problem with DC is it is administered in blood by intravenous route only which is a painful route of administration and usually patient avoids this due to incompliance^18^. Second is the absorption rate of DZ is in general incomplete, slow and erratic because of its poor water solubility^19^. Third, DZ is unstable and sensitive to light. Fourth, DZ cause myelosuppression and its usage in the form of combinatorial therapy is further restricted by its short half-life. Same as other anticancer medicaments, it also shows non-specific toxic effect on normal cells^20^. One efficient method to minimize these shortcomings is targeted DZ drug delivery with encapsulation in nanocarriers into skin cancer cells^21^.

Nano-cream/semi-solid emulsion is the topical preparations which externally applied^22,23^. Nanocream can be formulated by high-energy techniques like ultrasound generators, high-pressure homogenizers or high shear stirring^24^. Usually, a nano-cream is very effective in cosmetics and personal care due to the small droplets size in the range of nanoparticle (100–600 nm)^25^, which allows cream to spread and deposit smoothly and uniformly onto the surface of skin and increases the effective release of drug active ingredients on the skin surface^26,27^. The cream consists of several drugs ingredients for different disease curing activities in a suitable semisolid base either hydrophilic or hydrophobic in nature^28^.

Due to its low water solubility, short shelf life as well as the intraperitoneal route of administration, current study designed for evaluation of nanocream for the treatment of melanoma. In this experiment, we evaluated the newly formulated Dacarbazine loaded nanoparticle (DZNP) and Dacarbazine loaded nanoparticle (DZNC) as a topical formulation for the treatment of melanoma.

## Results

### Physical characterization of nanocream

Prepared nanocream was physically white in color, semi-solid in the state, smooth in texture and homogenous in nature (Table 1).

**Table 1:** Physical characterization of nanocream.

Characterization Parameters	Observation
Colour	White
State	Semi-solid
Texture	Smooth
Homogenity	Homogenous
pH	6.7 ± 0.14
Spreadability	55.23 ± 3.13 g cm/sec, Non-Newtonian Behaviour
Viscosity	Idea character
Drug Content Determination	0.00164 gm of Drug/1 gm of cream

### Particle size and Zeta potential analysis

**Figure 1:**
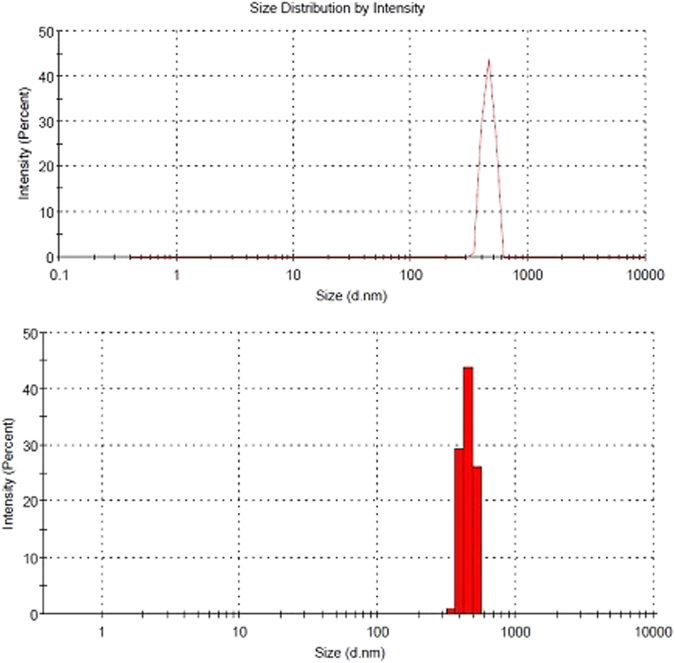
Nanoparticle size distribution by intensity. The experiment was repeated three times (Mean ± S D, n = 3).

**Figure 2:**
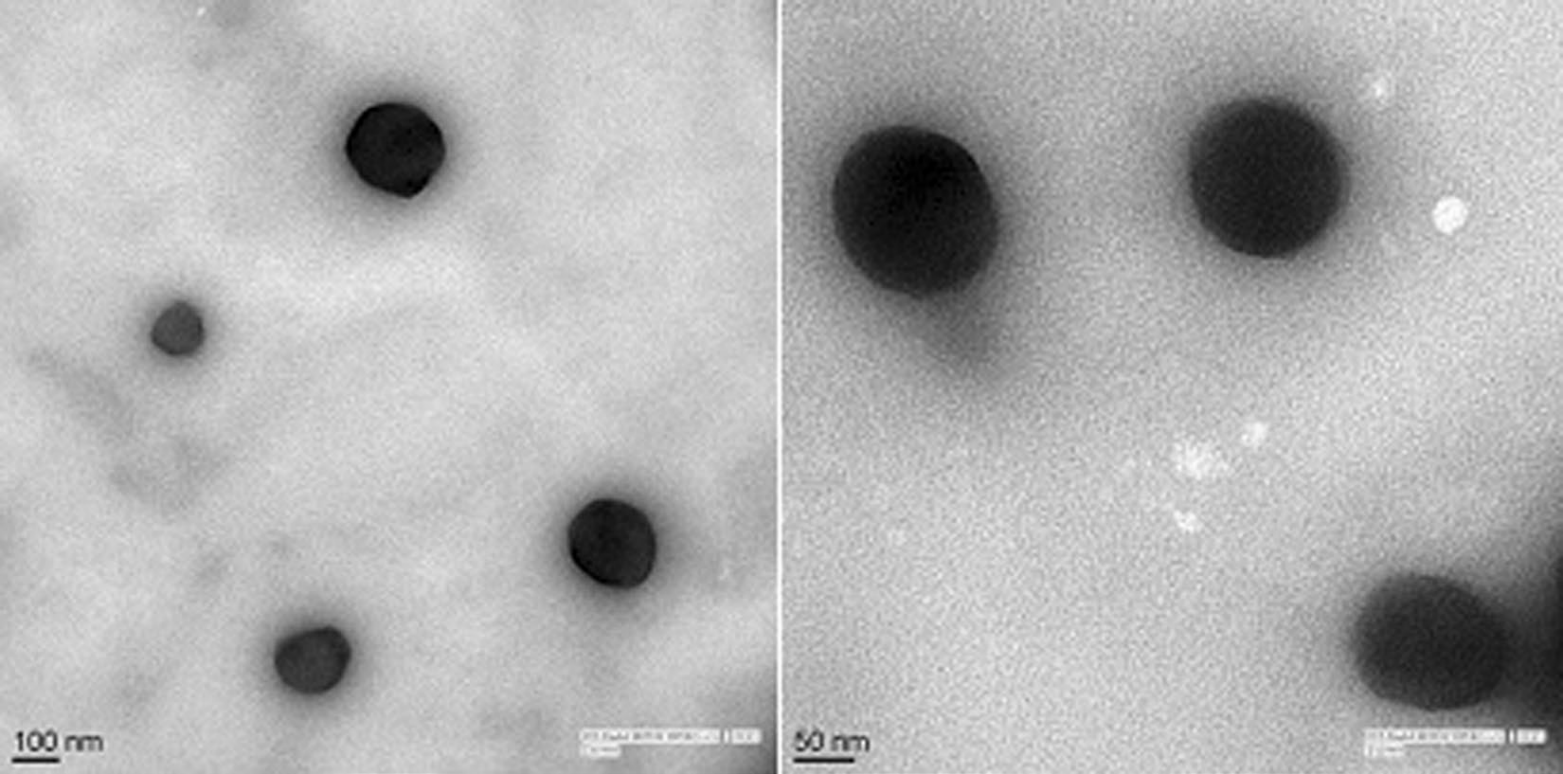
Transition Electron Microscopy (TEM) of nanoparticle. The experiment was repeated three times (Mean ± S D, n = 3).

Size of the particle us a key factor for the therapeutic potential of drug delivery in the form of nanoformulation. The size of the particle of DZNP was found to be 16.3 ± 8.1 nm (Figs 1 and 2). Similarly, the zeta potential of DZNP and DZNC (−3.51 ± 1.27) and (−5.63 ± 1.67) was also significantly reduced in comparison to dacarbazine suspension (−36.3 ± 1.84).

### TEM

The shape of the particle is a key factor for determination of the therapeutic potential of a nanoparticle formulation. Basic use of TEM was to characterize the surface morphology of nanoparticle formulations (Fig. 2). We found the uniform and spherical shape of DZNP and its cream without any change in the texture of the surface. The step of freeze-drying did not affect the texture of the surface of nanoparticle formulation.

### pH and spreadability

pH value of nanoparticle cream was found to be 6.7 ± 0.14. Spreadability value of nanocream was 55.23 ± 3.13 g cm/sec with non Newtonian behavior.

### Efficiency of nanoencapsulation and capacity of drug loading

Nanoencapsulation efficiency plays very crucial role in drug delivery by nanoparticle formulations. The nanoencapsulation efficiency of formulation was found to be 67.4 ± 3.5%. The capacity of Drug loading of the formulation found to be 6.73 mg/10 mg of nanoparticles.

### Viscosity analysis

**Figure 3:**
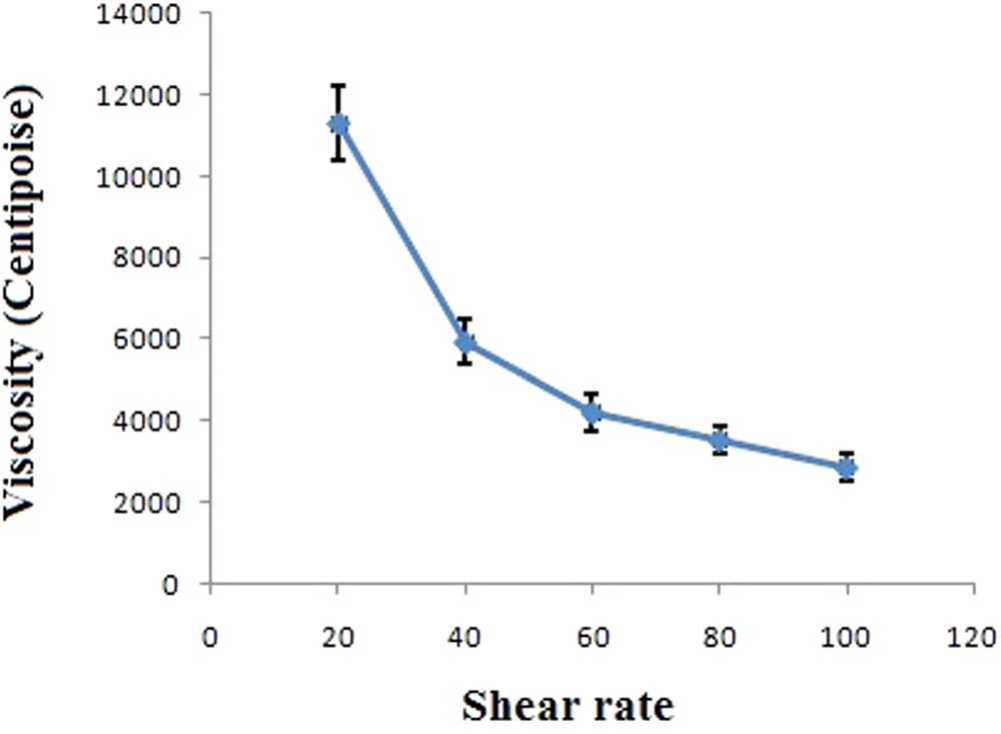
Viscosity of nanoparticle formulation. The experiment was repeated three times (Mean ± S D, n = 3).

The viscosity of nanoparticle formulation was measured with respect to shear rate. On low shear rate viscosity was high. With an increase of shear rate viscosity decreased which is depicted in Fig. 3.

### ***In vitro*** drug release

**Figure 4:**
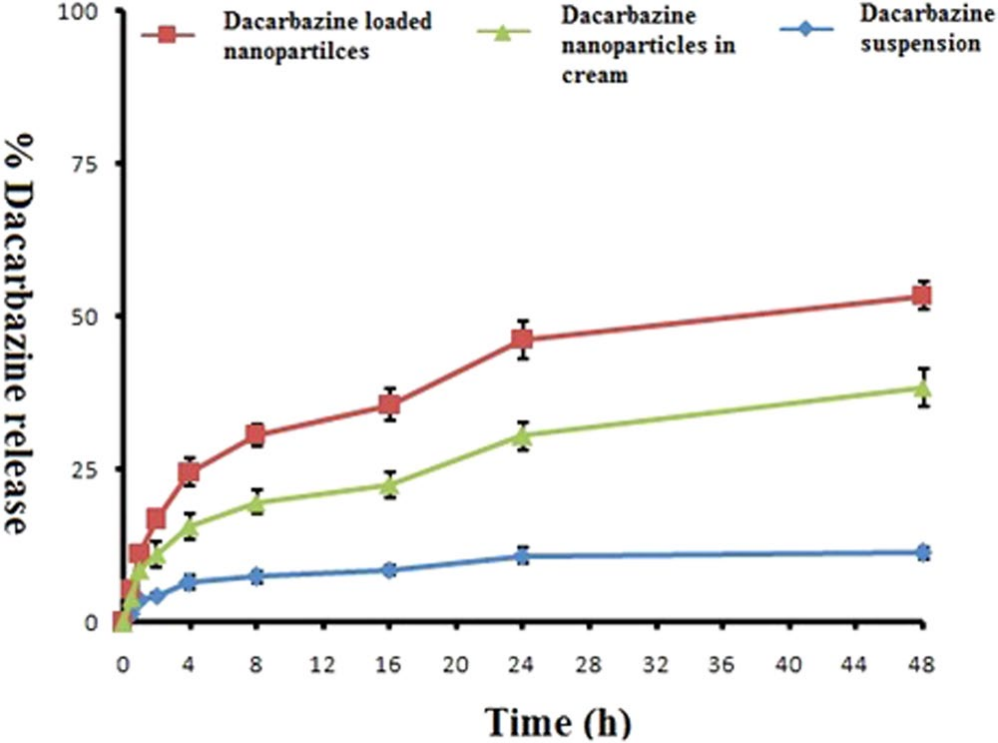
*In vitro* drug release of dacarbazine loaded nanoparticles and their formulation. The experiment was repeated three times (Mean ± S D, n = 3).

Franz diffusion cells method applied for the determination of release of drug from nanoparticle formulations. In this study, we compared percentage rate of release of drug in between drug-loaded nanoparticle, nanoformulation cream, and the drug suspension. Percentage drug release rate was higher in both DZNP and DZNC as compare to the drug suspension. Percent release of dacarbazine suspension, DZNP as well as DZNC was calculated and presented in Fig. 4.

### Cytotoxicity: MTT assay

**Figure 5:**
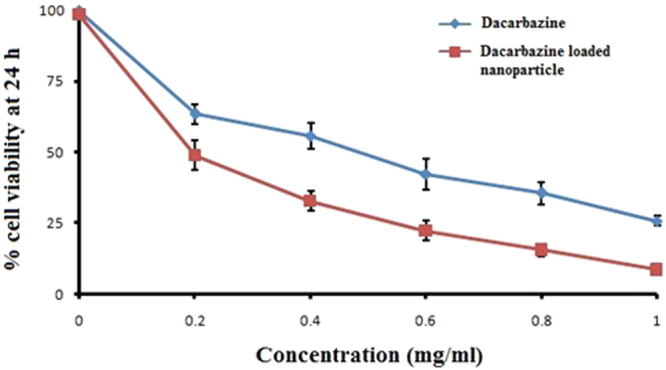
Cytotoxicity potential of dacarbazine loaded nanoparticle. The experiment was repeated three times (Mean ± S D, n = 3).

**Figure 6:**
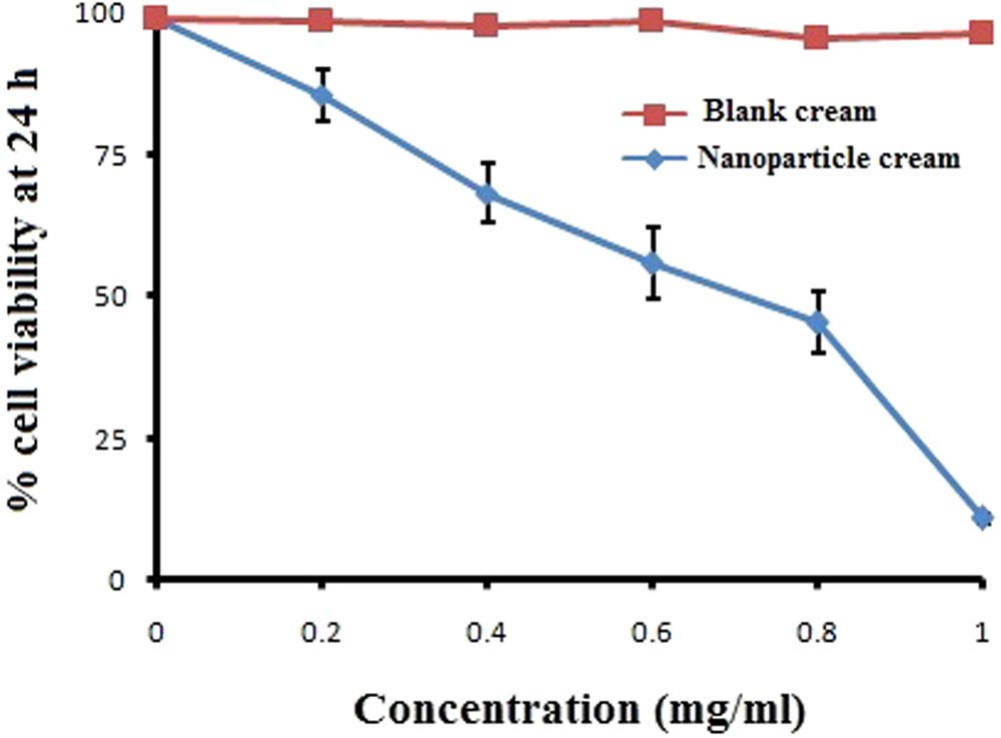
Cytotoxicity potential of dacarbazine loaded nanoparticle cream formulation in comparison to blank cream. The experiment was repeated three times (Mean ± S D, n = 3).

The cytotoxicity of DZNP and its cream formulation was measured against B16F1 mouse melanoma cancer cell line and expressed in terms of IC50 (drug concentration required for killing 50% of the alive cells) value. IC50 of dacarbazine was 0.48 mg/ml. IC50 value of DZNP was 0.19 mg/ml while 0.63 mg/ml for DZNC (Figs 5 and 6).

## Discussion

In this study, we have integrated dacarbazine in nanostructured lipid particles by using the technique of employing the solvent structured method^29^. Dacarbazine is poorly soluble in water. Due to the high solubility of dacarbazine in lipid, in the current study, we prepared dacarbazine nanocream in spite of gel. Further, cream retains on the skin for a longer time as compared to gel^30^. The cream was prepared from this nanoparticles. Dacarbazine is poorly soluble in water, has stunted half-life in blood circulation along with low responsiveness and more side effects^31^. Considering these characteristics of dacarbazine, the current study was designed for the dacarbazine loaded nanoparticles and its formulation prepared for topical delivery. As investigated by Moldovan and their colleagues, stability and durability are the outcomes of consistency in the dosage form of any formulation^32^. In our study, DZNC was found to be homogenous, smooth along with appropriate pH and spreadability which indicate durability and stability of the prepared formulation.

Morphology and particle size and its distribution play preliminary role in the evaluation of nanoparticulate formulations^33^. With the help of transition electron microscopy it is now very easy to characterize the morphology and size of nanoparticles in formulations^34^. Particle size in nanoformulations has a significant effect on the release of the drug. Smaller the nanoparticle size greater the surface area, which leads to quick drug delivery. Drugs loaded in nanocarrier when comes in contact with the surface area of the body causes significant release of the drug^35^. Passive targeting, as defined by Allegra and coworkers is the elevation in the permeability and capacity of retention through tumor vessels which is attributed to the minute size of the nanoparticle, which in turn allows the nanoparticles to escape from renal exclusion and reticuloendothelial system^36,37^. In our experiments, DZNP particle size was 16.3 ± 8.1 nm and these particle sizes were optimum for infiltration in skin melanoma cells. It may also aid endocytosis by crossing over the tissues due to its small size^38^.

The surface charge of the nanoparticle determines the interaction with the surrounding biological environment and their electrostatic interaction with biologically active compounds^39^. Charge present on the surface of the particle stipulated the stability of the formulation of nanoparticles by preventing the aggregation and subsequent collapse^40^. The difference between surface charges is determined by zeta potential and those of opposite signs are derived from the medium surrounding the particle. Zeta potential is an indirect index of the surface charge of the particle^41^. The colloidal stability is greater when zeta potential is more positive or negative. In general, optimum colloidal stability is arrives with zeta potentials of +/−40–50 mV^42^. Zeta potential of DZNP and DZNC was found to be (−3.51 ± 1.27) and (−5.63 ± 1.67) respectively. We witnessed a negative zeta potential, depending upon the composition of nanoformulation, which might be due to the presence of –COO- group in stearic acid. Moreover, the zeta potential is efficiently diminished to nearly neutral values when the drug is further loaded into the nanoformulation^43^. Hence, we predicted a high permanence and efficacy in the prepared dosage form.

Nanoencapsulation is one of the novel technique which allows the engulfment of the drug in a suitable carrier at the nano scale, and thus protects the core material, i.e. therapeutic drug. It is a coating of various drug particles within another material at the different the nanoscale size. The material which is encapsulated known as the internal phase. The encapsulation material is referred as the external phase^44^. It allows the drug release as and when required, in brief, it gives us better control of releasing the drug at the targeted site^45^. The efficiency of our formulated nanoencapsulation was estimated to be 67.4 ± 3.5%, which implied that major amount of Dacarbazine was encapsulated in the form of a nanoparticle. It is important to determine the amount of drug that can be loaded into the nanoparticles when lipid nanoparticles are being used as the drug carrier system. Drug loading capacity is the percentage of drug entrapped or loaded in nanocarrier^46^. Our nanoformulation had a drug loading capacity of 6.73 mg per 10 mg of nanoparticles which specifies that more than 67% of the drug was loaded in the nanocarrier which exhibits the appreciable loading capacity of our formulation.

TEM is a novel technique which can be exploited to observe the structure, crystallization, morphology, and stress of very small particles. It is a vital tool of characterization for direct imaging nanoparticles for quantitative measurements of particle morphology, size and its distribution^47^. TEM has been trending since its invention because of the limitation of scanning electron microscopy, which can barely provide data related to the morphology of nanoparticles^48^. The observation of particle size and TEM images substantiate that our nanoparticles and nanoformulations were spherical in shape, generally in the range of 10–20 nm. This range of particle size is compatible for penetration inside the cancerous cell. pH of human skin is around 5.5 which is slightly acidic^49^. pH of pharmaceutical formulation applied for the treatment of skin cancer must be compatible with pH of the skin. pH of our nanoformulation is 6.7 ± 0.14 which quite close and compatible with pH of skin.

Viscosity and the combination of other rheological factors determine the spreadability of creams. Additionally, Spreadability is an important property in the evaluation of semisolid formulations used for application through topical mucosal routes. It is a key factor for the overall performance of a cream formulation, hence it is carefully taken into consideration and effectively measured during the formulation development stages of cream^50^. Spreadability is the result of structural and viscoelastic physiognomies that portray the rigidity, strength along with the relative contributions of elastic and viscous behavior^32^. The ability of a semisolid formulation to spread on the skin plays a vital role in the administration of a standard medicated formulation and thus in the efficacy of a topical therapy^51^. In this study, spreadability of our nanoformulation was 55.23 ± 3.13 g.cm.sec^−1^, along with non-Newtonian behavior which shows our cream spreads 55.23 ± 3.13 g cm on the skin in a second. It reflects the ideal value for the skin spreadability of our nanoformulation. These results support the notion that our cream has significant spreadability.

Fluids of every kind have a fixed proportion of resistance towards any change of form whereas the solids exhibit a gradual yielding for the forces tending to change their structural form^52^. Generally, topical creams have high viscosity at low shear rates and vice versa, which means topical creams are non-Newtonian in nature. Therefore, it is essentially advisable that estimation of viscosity should be performed at shear rates that are equivalent to the situation we are trying to simulate^53^. Our nanocream shows the ideal characteristic of viscosity. Al low shear rate cream was highly viscous and viscosity decreases with the increase of shear rate.

The effectiveness of any nanoformulation is described by the rate of drug release. After topical application of the nanocream, the carrier must release the active moiety before it gets to contact the epidermal surface and hence is available for penetration into the stratum corneum and subsequent lower layers of the skin^54^. In our experiment nanoparticle as well as nanoformulation cream rate of drug release in higher as compared to the suspension of the drug. On the basis of drug release rate, this dacarbazine nanoparticle cream can be utilized for effective treatment of melanoma.

Cellular metabolic activity can be measured by the MTT assay, which is a colorimetric assay.

In this assay a yellow coloured salt of tetrazolium (3-(4,5-dimethylthiazol-2-yl)-2,5-diphenyltetrazolium bromide) also known as MTT reduced to purple colour which gives index of cellular metabolic activity which ultimately gives idea about the viability of cell^55^. NADPH dependent oxidoreductase enzymes are present in viable cells which reduce yellow MTT reagent to an insoluble crystalline product formazan which is deep purple in colour^56^. Crystals of formazan are then solubilized by dissolving it in solution and absorbance is recorded at 500–600 nanometers by using a plate reader. Colour of solution indicates the percentage of viability of cells. Darker the solution, the greater the number of metabolically active viable cells^57^. Hence, the metabolic activity measured by MTT assay is considered as an index of the proliferation of cancerous cells under investigation^58^. The proliferation of cancer cells is considered to higher if the metabolic activity is relatively higher than normal^59^. In our research, cell viability (%) was found to be less in DZNP as compared to the simple drug dacarbazine which indicates DZNP is more effective and suitable dosage form for drug delivery in melanoma as compare to dacarbazine. Furthermore, in comparison to blank cream DZNC shows an excellent cytotoxic effect on melanoma cells which indicates DZNC is the most suitable drug delivery form for the treatment of melanoma.

## Material and Methods

### Chemicals

Dacarbazine was purchased from TCI Fine Chemical Limited, Chennai (India). DMSO and Stearic acid were procured from Sigma Aldrich Company of USA. Water of HPLC grade, Acetonitrile, sodium-heptane sulphonate and methanol were purchased from Qualigens, Mumbai, India. Chemicals utilized in the experiments were of analytical grade and utilized without any purification.

### Cells and reagents

B16F1 cancer cell line was purchased from National Centre for Cell Science (NCCS), Pune. Mouse melanoma cell line B16F1 was kept in CO_2_ (5%) and air (95%) at 37 ◦C by using Dulbecco-Modified Eagle Media (Biological Industry, Israel) which was supported with serum of fetal calf (5%). All the experimental work was started with asynchronous populations in phase of exponential and rapid growth, 24 hour after the plating of sample^60^.

### Preparation of dacarbazine loaded stearic acid based nanoparticles

#### Procedure

Dacarbazine loaded stearic acid nanoparticles were prepared by the solvent diffusion method. In brief, dacarbazine (100 mg) and stearic acid (100 mg) was suspended in 6 ml of acetone and 6 ml of ethanol mixture which is known as organic-phase. Next, 120 ml of distilled water was heated up to 70 °C on a magnetic stirrer. This is called aqueous phase. The organic phase was then mixed into the aqueous-phase and mixing was continued for next 30 min. After cooling the turbid suspension at the normal room temperature, then the mixture was lyophilized in a freeze dryer (Lark Technology, New Delhi).

### Characterization of nanoparticles

#### Efficiency of nanoencapsulation and capacity of drug load

The efficient of nanoencapsulation and capacity of drug loading were measured by suspending of 10 mg dacarbazine sample loaded nanoparticles in 10 ml of DMSO, mixed properly and left aside for ten days period. Then after, treated sample was subjected to centrifugation at 40,000 rpm (Sorvell-ultracentrifuge) for the period of 1 h. Then supernatant centrifugated liquid was diluted as appropriate and was filtered out by 0.22 -µm membrane-filter. The filtrate absorbance was analyzed at 323 nm by the use of a UV spectrophotometer of Shimadzu, UV 1800, Kyoto, Japan model. The experimental work was repeated in triplicate times (n = 3). The efficiency of nanoencapsulation and capacity of drug load was measured by the use of the following formula:

$${\rm{Nanoencapsulation}}\,{\rm{efficiency}}:{\rm{Entrapped}}\,{\rm{drug}}\,{\rm{amount}}/{\rm{Added}}\,{\rm{drug}}\,{\rm{amount}}\times 100$$

$${\rm{Drug}}\,{\rm{loading}}\,{\rm{capacity}}:{\rm{Entrapped}}\,{\rm{drug}}\,{\rm{amount}}/{\rm{Nanoparticle}}\,{\rm{yield}}$$

#### Preparation of dermal cream containing dacarbazine loaded lipid nanoparticles

The pharmaceutical dermal cream of DZNP was prepared by oil-in-water emulsion technique. In brief, the required quantity of lipid soluble components, as specified in the table (Table 2), were taken in a china dish and mix together by melting on a hot plate. Subsequently, water soluble components were taken in another china dish and heated on the hot plate. The melted organic phase was then mixed to aqueous phase with regular mixing and stirring was continued in a uniform direction to form oil-in-water emulsion based dermal cream of DZNP.

**Table 2:** Composition of nanocream.

S. No.	Ingredients	Quantity (g)
1	Stearic acid	1.4296 g
2	Cetosteryl alcohol	0.15 g
3	Octadecanol	0.05 g
4	Potassium hydroxide	0.1 g
5	Sodium hydroxide	0.01 g
6	Polysorbate 80	0.4 g
7	Glycerine	4.2 g
8	Benzyl alcohol	0.1 ml
9	Water	6.5 ml
10	Propylene glycol	2.75 g
11	Dacarbazine Nanoparticles	0.0704 g

#### Size of Particle and Zeta potential assay

The diameter of the particle, index of polydispersity, and zeta potential of the DZNP and DZNC were measured by a laser-light-diffraction system by the use of a Mastersizer (ZEN3600 Nano-ZS model, Malvern Instruments Ltd., United Kingdom). Then samples were mixed and diluted with deionized cold water and analyzed at 4 °C. Data were measured by use of automatic Mastersizer-software provided by the manufacturer (Malvern Instruments Limited., United Kingdom). Experiments were carried out three times (n = 3).

#### Transmission electron microscopy (TEM)

The surface structure of nanoformulations was measured by the use of TEM (FTI-Tecnai-F20 model). Nanoformulation aqueous suspension was drop cast onto a grid which was carbon coated and then dried in air at the room temperature before the load into the microscope and maintained at an 80 kV voltage.

#### Determination of pH

pH meter was calibrated by the use of a standard solution of the buffer. Approximate 0.5 g cream was taken and mixed with distilled water (50 ml) and pH was calculated in triplicate (n = 3).

#### Determination of spreadability

For the measurement of spreadability, 3 g of sample was applied between two slides of glass and then pressed to uniform thickness by putting 1000 g load for five minutes. Then 100 g weight was added to the pan and the top plate was pulled by the help of string which attached to the hook. Time taken in which the slide of upper glass moves over to the lower plate was taken as a measure of spreadability. Short interval means the better spreadability. The spreadability (S) can be measured by the use of the formula

$${\rm{S}}={\rm{m}}.{\rm{l}}/{\rm{t}}$$

where, S: Spreadability; m: Weight tied to slide of upper glass; l: Moved length on glass slide; t: Taken time.

#### Determination of Viscosity

The viscosity of cream sample was measured by using Brookfield rheometer (Brookfield DV-3T + Rheometer) using an LV-4 spindle. In brief, the cream was poured into the adaptor of rheometer and viscosity of the test sample was determined as a function of the rate of the shear according to the standard operating procedure provided by the manufacturer.

#### Drug release *In vitro*

Drug uptake of skin and trans-epidermal permeation were measured with vertical Franz-diffusion cells kept in an incubator (34 ± 1 °C), with the compartments held together by a clamp. Thin section of the epidermis of porcine, isolated by the use of an Aesculap and treated same as previously published report^61^. 250 µL of an adequate sample of DZNP and DZNC was applied on the surface of the skin and with care, it was spread on the surface for uniformly with an area of diffusion 1.60 cm^2^. The medium of receptor consisted in a buffer of phosphate solution with 6.5 pH was regularly mixed by the use of a magnetic stirrer bar in the entire period of this study. Proper samples were withdrawn for the determination of Dacarbazine at regular intervals of time (1–24 h) and the cell was quickly re-filled with the solution of a fresh receptor. At the end of 24^th^ h of the experimental protocol, the site of application of the skin was washed out with the solution of normal saline of composition 0.9% w/v of sodium chloride, in order to remove the residual formulation on the surface. The skin was then segmented into short pieces with a scalpel and 2.0 mL of a phosphate buffer (pH 6.5)/DMSO (90/10 v/v), the mixture was added for Dacarbazine extraction. After 24 h of magnetic stirring at room temperature, the resulting suspension was centrifuged and the supernatant was analyzed by HPLC apparatus (Shimadzu), employing an UV-Vis detector fixed at 301 nm, a Purospheres Star RP-C18 end-capped column (Merck, 150 × 4.6 mm; 5 µm) and a mixture of 0.05 M 11 ammonium acetate/acetonitrile (90/10 v/v) at rate of flow of 1 mL/min as eluent. Skin uptake was expressed as Dacarbazine amount vs skin diffusion area (µg/cm^2^). The experimental study was repeated three times (n = 3).

#### Cytotoxicity: MTT assay

Standard cell proliferation assay was employed to detect the cytotoxicity of tailored cream and NPs in mouse melanoma cell line, B16F1. In brief, 7 × 10^3^ B16F1 cells were suspended in serum DMEM (200 μl) placed in each well of a 96 wells microtitre plate. The medium was substituted with serum-free DMEM after 24 h of the period of incubation. Following this, B16F1 cell line were incubated with a gradient concentration of Dacarbazine, DZNP, DZNP suspended in cream and blank cream prepared in a mixture of PBS (pH 7.4 + DMEM) corresponding to 0.2–1 mg/ml of dacarbazine for 24 hrs and 72 hrs respectively. In last, 5 mg/ml of MTT was put into an individual well of the microtitre plate and was incubated for next 4 hour at 37 °C. The crystals of formazan were formed after the lysis of the cell and were dissolved by the use of DMSO (100 μl). The absorbance was recorded at 570 nm and 630 nm as reference wavelength by ELISA reader (Tecan, Switzerland). Results were expressed in terms of IC_50_ value. IC_50_ refers to the drug concentration needed to kill 50% of the cells. Measurements were carried out three times (n = 3).

#### Statistics

Results were expressed inform of mean ± SD for n = 3 by applying statistics of the column in Graph Pad Prism-04. Unpaired t-test was applied for calculation of the statistically significant the difference in between mean values of various groups. Two ways ANOVA with Bonferroni-Post test was used for the calculation of difference of the statistical significance in between concentration of plasma. A value of P < 0.05 as a difference significant level was considered.
